# Expression of Rb2/p130 in breast and endometrial cancer: correlations with hormone receptor status

**DOI:** 10.1054/bjoc.2001.1923

**Published:** 2001-08

**Authors:** K Milde-Langosch, C Goemann, C Methner, G Rieck, A-M Bamberger, T Löning

**Affiliations:** Department of Gynecopathology, Institute of Pathology, University Hospital Eppendorf, Hamburg, Germany

**Keywords:** retinoblastoma protein family, oestrogen receptor, progesterone receptor, mammary carcinoma, endometrial carcinoma

## Abstract

Rb2/p130 is a member of the retinoblastoma family of proteins, consisting of Rb, Rb2 and p107, which are important negative regulators of cell cycle progression and differentiation. While Rb2 downregulation was observed in several malignant tumours including endometrial cancer, the role of p130 in breast carcinomas is still unknown. We investigated Rb2 protein expression in tumour tissue from 68 mammary and 41 endometrial carcinomas, 4 mammary cell lines, and normal tissue samples. Therefore, we performed Western blot experiments for Rb2, Rb, and the oestrogen and progesterone receptors (ER, PR-A, PR-B). Weak or absent Rb2 expression was more often found in endometrial (59%) than in mammary carcinomas (24%). We found significant positive correlations of Rb2 expression with Rb, ER, and PR-B in breast cancer samples, and of Rb2 with Rb, PR-A, PR-B, and younger age in endometrial carcinomas. No significant associations with histological grading, stage, nodal involvement, or Ki67 staining were detected. Rb2 mRNA expression was studied by semi-quantitative RT-PCR in 56 endometrial or mammary tissue samples and correlated significantly with Western blot results. Our results indicate that loss of Rb2 expression, mostly by transcriptional down-regulation, may be associated with the development and dedifferentiation of most endometrial and a subset of mammary carcinomas. © 2001 Cancer Research Campaign http://bjcancer.com


*British Journal of Cancer* (2001) **85**(4), 546–551
© 2001 Cancer Research Campaign
doi: 10.1054/ bjoc.2001.1923, available online at http://www.idealibrary.com on IDEAL
http://www.bjcancer.com

### Expression of Rb2/p130 in breast and endometrial cancer: correlations with hormone receptor status

**K Milde-Langosch, C Goemann, C Methner, G Rieck, A-M Bamberger and T Löning**

Institute of Pathology, Department of Gynecopathology, University Hospital Eppendorf, Hamburg, Germany

**Summary** Rb2/p130 is a member of the retinoblastoma family of proteins, consisting of Rb, Rb2 and p107, which are important negative regulators of cell cycle progression and differentiation. While Rb2 downregulation was observed in several malignant tumours including endometrial cancer, the role of p130 in breast carcinomas is still unknown. We investigated Rb2 protein expression in tumour tissue from 68 mammary and 41 endometrial carcinomas, 4 mammary cell lines, and normal tissue samples. Therefore, we performed Western blot experiments for Rb2, Rb, and the oestrogen and progesterone receptors (ER, PR-A, PR-B). Weak or absent Rb2 expression was more often found in endometrial (59%) than in mammary carcinomas (24%). We found significant positive correlations of Rb2 expression with Rb, ER, and PR-B in breast cancer samples, and of Rb2 with Rb, PR-A, PR-B, and younger age in endometrial carcinomas. No significant associations with histological grading, stage, nodal involvement, or Ki67 staining were detected. Rb2 mRNA expression was studied by semi-quantitative RT-PCR in 56 endometrial or mammary tissue samples and correlated significantly with Western blot results. Our results indicate that loss of Rb2 expression, mostly by transcriptional down-regulation, may be associated with the development and dedifferentiation of most endometrial and a subset of mammary carcinomas. © 2001 Cancer Research Campaign http://bjcancer.com

**Keywords**: retinoblastoma protein family; oestrogen receptor; progesterone receptor; mammary carcinoma; endometrial carcinoma

The members of the retinoblastoma gene family, Rb/p105, Rb2/p130 and p107, play a central role in the regulation of cell cycle progression and differentiation in mammalian cells. In their active, hypophosphorylated form, these 'pocket proteins' bind and inactivate transcription factors of the E2F family (E2F-1 to E2F-5) which are important regulators of genes involved in DNA synthesis and mitosis (Mulligan and Jacks, 1998). The prototype protein of this family, Rb/p105, forms complexes with E2F-1, -2, and -3 in the G1 phase of the cell cycle. Phosphorylation of Rb in late G1 by cyclin-dependent kinases releases E2F transcription factors and allows progression into S phase (Weinberg, 1995). In addition to this function, Rb also acts as a transcriptional repressor which interacts with other transcription factors like c-jun, c/EPB, SP1, or E2F modulating their repressor or activator function (Udvadia et al 1993; Mulligan and Jacks, 1998). Somatic mutations of the Rb gene were found in sporadic retinoblastomas, lung carcinomas, osteosarcomas, breast carcinomas (7–19%) etc. (Weinberg, 1995). The role of Rb2/p130 in human cancer is less well studied. Hypophosphorylated Rb2 proteins bind to E2F-4 and E2F-5 in G0 and early G1 phase of the cell cycle, whereas the inactive, hyperphosphorylated form predominated in the S phase (Mulligan and Jacks, 1998). In some experimental systems, Rb2 is an effective tumour suppressor (Howard et al, 1998; Pupa et al, 1999). Rb2 mutations in human tumours have been found only sporadically in nasopharyngeal and lung carcinomas (Claudio et al, 1994, 2000). Rb2 protein expression has been studied in lung cancer (Baldi et al, 1996; Helin et al, 1997) and oral carcinomas (Tanaka et al, 1999) showing an inverse correlation with grading in both tumour types, and in malignant lymphomas, where it was associated with high levels of the proliferation marker Ki67 (Leoncini et al, 1999). In endometrial carcinomas, Susini et al (1998) found an association of low Rb2 expression with higher age, aneuploidy, high grading, and an unfavourable prognosis. Frequent deletions of chromosome 16q12.2, the locus of the human Rb2 gene, suggest that Rb2 might also be involved in mammary carcinomas (Yeung et al, 1993).

In the present study, we investigated Rb2 protein expression in 68 mammary carcinomas, 4 mammary cell lines, 41 endometrial carcinomas, and some normal tissue samples by Western blot experiments, followed by densitometric quantification of band intensities. In addition, Rb2 mRNA expression was analysed in 35 endometrial and 21 mammary tissue samples. We found associations of Rb2 expression with Rb and hormone receptors in both tumour types. Comparison of RT-PCR and Western blot results and the band pattern observed in immunoblots suggest that Rb2 expression might be predominantly regulated at the level of transcription.

### MATERIALS AND METHODS

### Tissue collection

All patients were treated at the Hamburg University Hospital, Dept. of Obstetrics and Gynecology, throughout 1994–1999. All tissue samples were carefully dissected immediately after surgery and stored in liquid nitrogen.

#### *Mammary carcinomas*

We studied tumour specimens from 68 breast cancer patients (mean age 56.2 years; range 30–85 years) and tumour-free mammary tissue from 7 patients. Histologically, 51 carcinomas

*Received 22 December 2000*
*Revised 24 April 2001*
*Accepted 30 April 2001*

*Correspondence to:* K Milde-Langosch were ductal and 12 cases were lobular tumours. In addition, 4 mucinous and 1 medullary carcinoma were analysed. 7 tissue samples were from recurrences, and 61 from primary tumours. 27 of the primary carcinomas were nodal-negative, whereas 33 tumours were nodal-positive, and nodal involvement is unknown in one case. The pathological staging of the primary tumours was done as recommended by the UICC. 12 tumours were classified as stage 1, 30 cases as stage 2, 4 tumours as stage 3 and 9 tumours as stage 4, whereas staging data were not available in 6 cases. According to histological examination, 3 specimens were highly differentiated (G1), 34 tumors were classified as G2, and 31 cases were high-grade carcinomas (G3).

### *Endometrial carcinomas*

41 endometrial tumours (mean age 64.7 years; range 39–83 years) and normal endometrial samples from 9 tumour patients were studied. Among the carcinomas, 14 cases were classified as FIGO stage 1a, 14 tumours as stage 1b, 5 cases as stage 1c, 3 cases as stage 2b and 1 tumour as stage 3. In 4 cases, staging data were not available. Histologically, 28 tumours were of the endometrioid type. In addition, 5 adenosquamous carcinomas, 1 serous-papillary tumour, 1 clear cell carcinoma, 2 tumours of mixed differentiation, 2 undifferentiated carcinomas and 2 carcinosarcomas were analysed. 17 carcinomas were highly differentiated (G1), 14 tumours showed moderate differentiation (G2), and 10 tumours were poorly differentiated (G3).

### Cell lines

The mammary carcinoma cell lines T47D (receptor-positive) and MDA-MB231 (receptor-negative) and the mammary epithelial cell line HBL-100 were purchased from from ATCC, Rockville, MD and cultivated as described (Bamberger et al, 1999). The receptor-positive MCF-7 breast cancer cells were obtained from Dr Fritz Hölzel, University Clinics of Obstetrics and Gynecology, Hamburg.

### Western blot analysis

Protein extraction and Western blots were performed as described (Bamberger et al, 1999). Frozen tissue was minced and cells were lysed in ice-cold sample buffer b1 (50 mM Tris pH 6.8, 1% SDS, and 10% sucrose), and protein concentration was determined following standard protocols. For electrophoresis, samples were diluted with a 1:1 mixture of sample buffer b1 and b2 (containing 50 mM Tris pH 6.8, 3% SDS, 10% sucrose, 10% β-mercaptoethanol, and 0.01% bromphenol-blue) to a final volume of 100 µl and a final protein concentration of 400 µg $ml^{-1}$, and heated at 99 ˚C for 5 min. Equal amounts of protein (40 µg) of each tumour sample were loaded per well, and equal loading was verified by immunoblotting with actin antibodies. Electrophoresis was performed in a 6% (Rb2), 8% (Rb, PR), or 10% (ER, actin) polyacrylamide separating gel and a 3% stacking gel. Proteins were transferred to a polyvinylidene difluoride (PVDF) membrane (Immobilon P, Millipore, Eschborn, Germany). After overnight incubation at 4 ˚C in blocking solution (0.1 M maleic acid, pH 7.5, 0.15 M NaCl, 0.005% Thimerosal, and 1% blocking reagent, Boehringer Mannheim, Germany), membranes were incubated for 1 h at room temperature with the following antibodies at the indicated dilutions in 9:1 TBST/blocking solution: rabbit anti-Rb2 (C-20; Santa Cruz, 1:1000), mouse anti-Rb (G3-245, Pharmingen, 1:400), mouse anti-ER (NCL-ER-6F11, Novocastra, 1:1000), mouse anti-PR (NCL-PGR, Novocastra, 1:300), goat anti-actin (I-19, Santa Cruz, 1:10 000).

After washing, blots were incubated with the second antibody (peroxidase-conjugated anti-mouse-IgG, 1:2000, peroxidase-conjugated anti-rabbit-IgG, 1:5000, or peroxidase-conjugated anti-goat-IgG, 1:4000, all from Santa Cruz) for 1 h at room temperature. The second antibodies were visualized by chemiluminescence reagents (Super Signal West Pico kit, Pierce, Rockfort, Ill.) with Hyperfilm ECL films (Amersham, Braunschweig, Germany). In parallel experiments with the same antibody, 20 µg protein extract from a positive cell line (T47D or MCF-7) was always loaded in one well as control for comparable exposition of chemiluminescent membranes and as standard for densitometry (GS-700 Imaging Densitometer, BioRad, München, Germany). The intensities of the specific protein bands were calculated as percent intensity of the control sample and corrected for equal actin loading. For statistical analysis, the samples were divided into 2 or 3 groups with similar band intensities for each antibody.

### Immunohistochemistry

Ki67 antigen was detected in all mammary and 37 endometrial tumours with MIB1 monoclonal antibodies (Dianova, Hamburg, Germany), and the percentage of positive tumour cells was determined.

### RNA extraction and RT-PCR

Total RNA was extracted from 100–200 mg tissue specimens with an RNeasy midi kit (Qiagen, Hilden, Germany). 2.5 µg total RNA were transcribed to cDNA using oligo-dT primers and MMLV reverse transcriptase (Life Technologies, Eggenstein, Germany). PCR was performed with primers from exon 6 (p130–67F: CTCACTGGTTTCTAGAACC) and exon 7 (P130-RR: CGTTCAGACACCTTGAGAGAG) with an annealing temperature of 59˚C yielding amplication products of 160 bp. After electrophoresis in 3% agarose gels and ethidium bromide staining, Rb2 mRNA expression results were evaluated in a semi-quantitative way as negative, weak, moderate, or strong. As control, a 240 bp fragment from actin mRNA was amplified as described (Schlott et al., 1997) which was always positive (Figure 3). Parallel samples without addition of reverse transcriptase were always negative (not shown).

### Statistical analysis

Correlations of the data were examined by $\chi^2$ statistics and Fisher's exact test using the SSPS 10.0 software. *P* values of 0.05 or less were considered statistically significant.

### RESULTS

### Rb2 expression in mammary carcinomas

Rb2 expression was found in all mammary-derived cell lines (Figure 1A), with the weakest expression found in T47D cells. Differences in electrophoretic mobility indicated phosphorylation of Rb2 proteins in MCF7 and MDA-MB231 cells, whereas T47D and HBL-100 cells predominantly showed a faster migrating band,

© 2001 Cancer Research Campaign

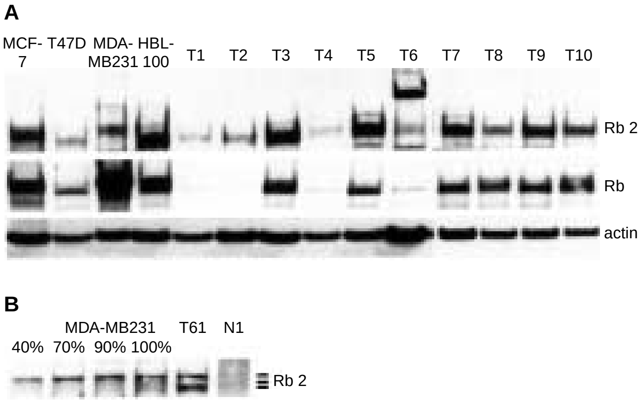

**Figure 1A** Expression of Rb2 and Rb in mammary cell lines and carcinomas. As loading control, the Rb2 blot was incubated with actin antibodies. (**B**) Rb2 expression in MDA-MB231 breast cancer cells in different states of confluency representing exponentially growing and growth-inhibited cells (20 µg protein applied per well). For comparison, one tumour and one normal mammary tissue sample are shown.

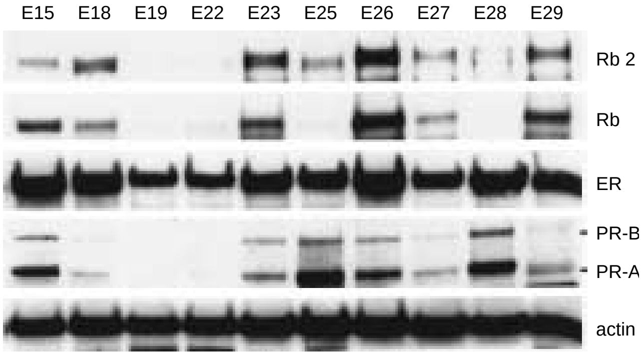

**Figure 2** Expression of Rb2, Rb, the oestrogen receptor (ER) and the progesterone receptor isoforms (PR-A, PR-B) in endometrial carcinomas

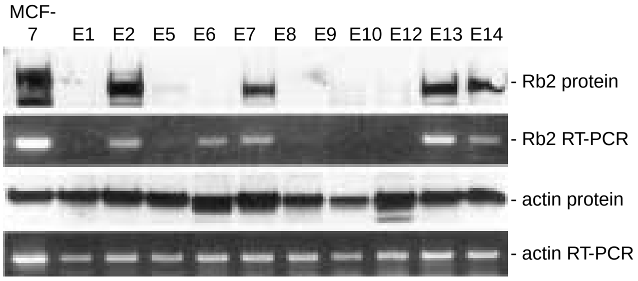

**Figure 3** Comparison of Rb2 protein expression as determined by Western blots (top) and Rb2 RNA expression as measured by RT-PCR (bottom) in 10 endometrial carcinomas and the mammary carcinoma cell line MCF7. As control, Western blot and RT-PCR results for a housekeeping gene are shown probably representing the hypophosphorylated, active form. Further examination of exponentially growing versus confluent MDA-MB231 cells showed increased Rb2 expression and the appearance of 2 bands of higher electrophoretic mobility in confluent cells (Figure 1B). In the clinical breast cancer samples, Rb2 protein was detectable in 65/68 (96%) cases under our experimental conditions. For comparison and relative quantification of Rb2 expression in different tissue samples and for similar exposition of chemiluminescent membranes, a control sample (MCF7 cell extract) was loaded on each gel. Compared with this 100%-standard, less than 20% Rb2 expression was found in 16 tumours (24%), moderate expression (20–50% of control) was observed in 28 cases (41%), whereas more than 50% was found in 24 carcinomas (35%). In all Rb2-positive tumour samples, the majority of Rb2 proteins was found in its faster migrating, hypophosphorylated form. In some cases, a second band of minor intensity with lower electrophoretic mobility was observed (tumors T3, T5, T7, T61; Figure 1A and B). In the 7 normal mammary tissue samples, Rb2 expression was low or moderate (mean 12.4% of control, range 1–33%; Figure 1B). Yet, because of the relatively low amounts of epithelial cells in these specimens, the results cannot be correlated with the Rb2 results in the corresponding carcinomas. In 2 tumours, a weak 130 kD-band was accompanied by a second, strongly immunoreactive band at ca 160 kD (see Figure 1A, tumour T6).

### Rb2 expression in endometrial carcinomas

Using the same cell line as control, Rb2 expression in endometrial carcinomas was generally lower than in breast cancer samples. Under the same experimental conditions, Rb2 was undetectable in 12/43 tumours (27%), and less than 20% Rb2 expression of the control cell line were found in 59% of the carcinomas (Table 2). As the case number was lower than in mammary tumours, only 2 groups with less than 20% expression or more were compared for statistical analysis. In normal endometrial tissue samples, Rb2 expression was similar to or weaker than in the corresponding tumour tissue (mean 22.5% of control, range 5–54%). Similar to the mammary carcinomas, only a small fraction if any of the Rb2 proteins was found in its hyperphosphorylated, inactive form (Figure 2). In none of the endometrial tumour samples, aberrant bands similar to those found in 2 breast cancer samples were found.

### Correlations of Rb2 expression with prognostic parameters and Rb expression in mammary carcinomas

Correlations with histological and clinical parameters, expression of the proliferation marker Ki67, steroid hormone receptor status, and Rb expression are shown in Table 1.

In mammary carcinomas, reduced Rb2 expression (< 20% of the standard) was more often found in lobular carcinomas (50% of the cases) than in the ductal type (18%; Table 1), but the small number of lobular tumours does not allow reliable correlations. No association of Rb2 results with age, clinical stage, nodal involvement, histological grading or expression of the proliferation marker Ki67 was observed.

The oestrogen receptor ER was detectable in all but 4 mammary carcinomas, all normal mammary tissue samples, and the cell lines T47D and MCF-7 in varying concentrations. ER results had been evaluated before in a semiquantitative manner as negative, weak, moderate or strong (Table 1). The progesterone receptor PR was undetectable under our experimental conditions in 47% (32/68) of the mammary carcinomas and in the cell lines MDA-MB231 and HBL-100 with known receptor-negative status. In PR-positive cases, the receptor was generally expressed in 2 isoforms: the bigger PR-B which is regarded as the active transcription factor

 *© 2001 Cancer Research Campaign*

**Table 1** Rb2/p130 expression in mammary carcinomas ($n$ = 68). Associations with histological parameters, hormone receptor status, Rb and Ki67 expression

| | | Rb2 expression[a] | | | | |
|---|---|---|---|---|---|---|
| | | *n* | < 20% | 20–50% | > 50% | *P* |
| Histological type | ductal | 51 | 9 | 26 | 16 | |
| | lobular | 12 | 6 | 1 | 5 | |
| | others | 5 | 1 | 1 | 3 | 0.033 |
| Grading | G1–2 | 37 | 9 | 13 | 15 | |
| | G3 | 31 | 7 | 15 | 9 | n.s. |
| ER expression[b] | weak/moderate | 31 | 14 | 10 | 7 | |
| | strong | 37 | 2 | 18 | 17 | 0.001 |
| PR-B expression[b] | negative | 37 | 13 | 16 | 8 | |
| | positive | 31 | 3 | 12 | 16 | 0.011 |
| PR-A expression[b] | negative | 32 | 10 | 13 | 9 | |
| | positive | 36 | 6 | 15 | 15 | n.s. |
| Rb expression[a] | <1% | 17 | 11 | 5 | 1 | |
| ($n$ = 60) | 1–10% | 14 | 2 | 10 | 2 | |
| | >10% | 29 | 1 | 9 | 19 | <0.001 |
| Ki67 expression[c] | <10% | 13 | 3 | 5 | 5 | |
| | 11–49% | 36 | 7 | 16 | 13 | |
| | ≥ 50% | 16 | 5 | 5 | 6 | n.s. |

[a]Relative to MCF7 control cells. [b]Relative to T47D cells. [c]Percentage of positive nuclei in tumour cells ($n$ = 65).

**Table 2** Rb2/p130 expression in endometrial carcinomas ($n$ = 41). Associations with histological parameters, hormone receptor status, Rb and Ki67 expression, and age of the patients

| | | Rb2 expression[a] | | | |
|---|---|---|---|---|---|
| | | *n* | < 20% | > 20% | *P* |
| Histological type | endometrioid | 28 | 18 | 10 | |
| | others | 13 | 6 | 7 | n.s. |
| Grading | G1 | 17 | 10 | 7 | |
| | G2 | 14 | 11 | 3 | |
| | G3 | 10 | 3 | 7 | n.s. |
| ER expression[b] | ≤150 | 24 | 16 | 8 | |
| | >150 | 17 | 8 | 9 | n.s. |
| PR-B expression[b] | <1 | 20 | 16 | 4 | |
| | >1 | 21 | 8 | 13 | 0.006 |
| PR-A expression[b] | <10 | 20 | 17 | 3 | |
| | >10 | 21 | 7 | 14 | 0.001 |
| Rb expression[a] | <10 | 16 | 13 | 3 | |
| | >10 | 25 | 11 | 14 | 0.018 |
| Ki67 expression[c] | <50% | 21 | 14 | 7 | |
| | ≥50% | 14 | 8 | 6 | n.s. |
| Age | <65 | 19 | 9 | 10 | |
| | >65 | 22 | 15 | 7 | n.s. |

[a]Relative to MCF7 control cells. [b]Relative to T47D cells. [c]Percentage of positive nuclei in tumour cells ($n$ = 35).

and the truncated PR-A whose function is not clear (Figure 2). In 5 mammary tumours, only a weak PR-A band without detectable PR-B expression was found. PR-A and PR-B expression were analysed separately with T47D cells as control. For statistical analysis, PR-positive and PR-negative tumours were combined.

In breast cancer samples, significant correlations of Rb2 with ER and PR-B expression were found (Table 1), whereas the association with PR-A was not statistically significant. These associations could not be observed in mammary cell lines: Rb2 expression was detectable in receptor-positive (T47D, MCF7) and receptor-negative cells (MDA-MB231, HBL-100) with different electrophoretic mobilities indicating hyperphosphorylation in MCF-7 and MDA-MB231 cells.

Expression of the retinoblastoma protein Rb was determined with MCF-7 or MDA-MB231 protein extracts as control. Rb was undetectable in 7/60 breast cancer samples (12%) under our experimental conditions. There were strong positive correlations of both retinoblastoma family members, Rb2 and Rb, in mammary carcinomas (Table 1). In contrast, no significant associations of the retinoblastoma protein Rb and steroid hormone expression were found (not shown).

### Correlations of Rb2 expression with prognostic parameters and Rb expression in endometrial carcinomas

Rb2 expression in endometrial carcinomas was not associated with histological type, histological grading, Ki67 staining (Table 2), or clinical stage (not shown). Reduced Rb2 expression (<20% of control) was more often found in older than in younger patients (68% versus 47%; Table 2). However, this difference did not reach statistical significance.

The oestrogen receptor ER was detectable in all endometrial tissue samples in varying intensities, whereas PR was found in 26/41 (63%) of the endometrial tumours. Small contaminations of normal endometrial tissue which always expresses steroid hormone receptors might have influenced the results for the tumours which, therefore, should be regarded with care. With T47D proteins as 100%-standard, 2 tumour groups of similar size with less or more than 150% of ER expression were combined for statistical analysis. For PR-A and PR-B, 2 groups of similar size with negative/low or stronger PR expression were compared. In contrast to mammary carcinomas, Rb2 expression was significantly associated with PR-B and PR-A, but not to ER expression in endometrial tumours (Table 2). In addition, there was a strong positive correlation of Rb2 and Rb.

Similar to Rb2, expression of the retinoblastoma protein Rb in endometrial carcinomas correlated significantly with PR-A ($P$ = 0.003) and PR-B ($P$ = 0.019), but to none of the other analysed parameters (not shown).

### Rb2 mRNA expression in endometrial and mammary carcinomas

Rb2 mRNA expression was analysed in a semi-quantitative way by RT-PCR with RNA derived from 29 endometrial carcinomas, 21 breast cancer samples, 6 normal endometrial tissue samples from tumour patients, and 3 mammary cell lines (T47D, MDA-MB231, and HBL-100). In normal endometrium, absent ($n$ = 4), weak ($n$ = 1), or strong ($n$ = 1) mRNA expression was detected. In endometrial carcinomas, Rb2 mRNA expression was absent ($n$ = 6), weak ($n$ = 8), moderate ($n$ = 6) or strong ($n$ = 9). Similar to our Rb2 Western blot results, Rb2 expression on average is higher in mammary than in endometrial carcinomas with weak ($n$ = 3), moderate ($n$ = 9) or strong expression ($n$ = 9). There was a good correlation of Western blot and RT-PCR results in both tumour types indicating that Rb2 expression is regulated at the level of transcription in the majority of the tumours (Table 3).

### DISCUSSION

Our results have shown that loss of Rb2 expression is a frequent event in endometrial carcinogenesis and correlates significantly

© 2001 Cancer Research Campaign 

**Table 3** Comparison of RT-PCR and Western blot results in 35 endometrial tissue samples and 21 breast cancer samples

| **Rb2 protein expression (western blot)**[a] | **Rb2 RNA expsression (RT-PCR)** Negative | Weak | Moderate | Strong | |
|---|---|---|---|---|---|
| endometrial tissue samples[b]: | | | | | |
| < 20 | 10 | 7 | 2 | 3 | |
| > 20 | – | 2 | 4 | 7 | *P* = 0.003 |
| breast cancer samples: | | | | | |
| < 20 | – | 2 | 2 | – | |
| > 20 | – | 1 | 7 | 9 | |
| all tissue samples | 11 | 12 | 15 | 20 | |

[a]Rb2 protein expression relative to the control; [b]29 carcinomas and 6 normal endometrial tissue samples from tumour patients.

with PR expression in these tumours. Failure of ER/PR expression is regarded as an important step in advanced endometrial carcinogenesis and was previously shown to correlate with poor prognosis, and with negative expression of Rb (Li et al, 1996). The close association of PR and Rb2 expression found by us indicates that loss of Rb and Rb2 might be involved in tumorigenesis. Decreased Rb2 expression often also coincided with lower ER expression, but this association did not reach statistical significance. Although this might be due to a relatively low sample size and heterogeneity of the group of tumours, our data clearly show that Rb2 expression correlated more strongly with PR-A and PR-B than with ER.

In a multivariate study including p130 expression, grading, stage, and ploidy of endometrial tumours, low Rb2 expression was an independent predictor of unfavourable outcome (Susini et al, 1998). Future studies will clarify if Rb2 is an independent prognostic indicator or if this effect is only due to the association with hormone receptor status. In addition, Rb2 expression was associated with younger age as shown before (Susini et al, 1998), which corresponds to the decrease of hormone receptor concentration in the endometrium with higher age.

Up to now, less is known about the role of p130/Rb2 in mammary carcinomas. Compared with endometrial tumours, Rb2 expression in these tumours was higher and showed less variation. In 2 cases, strong bands of higher molecular weight (see Figure 1, case T6) might be the result of underlying genetic abnormalities. In contrast to endometrial carcinomas, Rb2 was not only associated significantly with Rb and PR, but also with ER.

Although the mammary gland and the endometrium are both hormone-responsive, there are differences in the physiological role of ER and PR in these organs: in endometrial cells, ligand-bound ER promotes intensive proliferation in the first half of the menstrual cycle, whereas PR triggers terminal differentiation in the secretory phase. Endometrial carcinomas are nearly always ER-positive, but partly PR-negative (Satyaswaroop and Tabibzadeh, 1997). In mammary epithelial cells, the difference between ER and PR function is less clear-cut (Miller and Langdon, 1997). The proliferation is only weakly stimulated by ER, and loss of both receptors is observed during the processes of progression and dedifferentiation in many tumours. Taken together, Rb2 expression in both tumours is associated with hormone receptors involved in differentiation processes, namely PR in endometrial tumours and both ER and PR in mammary carcinomas.

Previous studies have shown that Rb is not only an important inhibitor of cell-cycle progression, but also involved in processes of differentiation (Weinberg, 1995). For Rb2, experimental data have shown that its expression is a prerequisite of granulocyte differentiation (Mori et al, 1999) and high Rb2 expression was found in terminally differentiated cells (Baldi et al, 1997). Our data support the view that Rb2 is involved in differentiation, at least in mammary and endometrial cells.

How can the close correlation of Rb2, Rb and hormone receptors be explained? As far as it is known, oestrogen- and progesterone-responsive elements (ERE, PRE) are not involved in the regulation of Rb and Rb2. A common feature of ER, PR, and Rb is the ability to interact with jun/fos dimers and activate gene expression at AP-1 binding sites (Nishitani et al, 1999). In addition, the expression of ER and Rb is regulated at least partly from AP-1 sites in their promoter or enhancer regions (Linardopoulos et al, 1993; Tang et al, 1997). In a previous study, we could show different patterns of AP-1 proteins in receptor-positive and receptor-negative mammary carcinomas (Bamberger et al, 1999). Although the precise mechanism is still unknown, these data suggest that AP-1 transcription factor complexes might be involved in the common regulation of Rb and hormone receptors, and possibly Rb2.

Cell-culture experiments revealed the presence of 3 p130 forms of different phosphorylation states and electrophoretic mobility: 2 faster migrating forms which predominate in G0/G1 and are able to bind the E2F-4 transcription factor, and 1 slowly migrating, more phosphorylated, inactive form which is the only detectable form in the S/G2/M phases of the cell cycle (Mayol et al, 1996). Similar to these results, one slowly migrating p130 form predominated in exponentially growing MDA-MB231 breast cancer cells in our study, whereas 2 additional bands of higher electrophoretic mobility probably representing the active repressor appeared in confluent cells. In the tumour tissues analysed in this study, only 2 Rb2 bands can be clearly distinguished, and the faster migrating, active form predominated in all cases, and in T47D and HBL-100 cells. These results indicate that phosphorylation is not a major way of Rb2 inactivation in endometrial and mammary carcinogenesis. In contrast, the corresponding results of protein and mRNA analysis in most cases indicate that down-regulation of Rb2 in these carcinomas might take place mainly on a transcriptional level.

Taken together, our results indicate that loss of Rb2 expression may be associated with the development and dedifferentiation of most endometrial and a subset of mammary carcinomas. Further studies will be necessary to clarify the role of p130 down-regulation in carcinogenesis and the interactions of retinoblastoma proteins and steroid hormone receptors in these tumours.

### ACKNOWLEDGEMENTS

We thank Bianca Kelp for her help with cultivation of the cell lines and Jochen Koppelmeyer for excellent photographical work. This work was supported by the Hamburger Stiftung zur Förderung der Krebsbekämpfung.

### REFERENCES

Baldi A, Esposito V, De Luca A, Howard CM, Mazzarella G, Baldi F, Caputi M and Giordano A (1996) Differential expression of the retinoblastoma gene family members pRb/p105, p107, and pRb2/p130 in lung cancer. *Clin Cancer Res* **2**: 1239–1245

Baldi A, Esposito V, De Luca A, Fu Y, Meoli I, Giordano GG, Caputi M, Baldi F and Giordano A (1997) Differential expression of Rb2/p130 and p107 in normal human tissues and in primary lung cancer. *Clin Cancer Res* **3**: 1691–1697

 *© 2001 Cancer Research Campaign*

Bamberger A-M, Methner C, Lisboa BW, Städtler C, Schulte HM, Löning T and Milde-Langosch K (1999) Expression pattern of the AP-1 family in breast cancer: association of fosB expression with a well-differentiated, receptor-positive phenotype. *Int J Cancer* **84**: 553–558

Claudio PP, Howard CM, Baldi A, De Luca A, Fu Y, Condorelli G, Sun Y, Colburn N, Calabretta B and Giordano A (1994) p130/pRb2 has growth suppressive properties similar to yet distinctive from those of retinoblastoma dmily members pRb and p107. *Cancer Research* **54**: 5556–5560

Claudio PP, Howard CM, Fu Y, Cinti C, Califano L, Micheli P, Mercer EW, Caputi M and Giordano A (2000) Mutations in the retinoblastoma-related gene RB2/p130 in primary nasopharyngeal carcinoma. *Cancer Res* **60**: 8–12

Helin K, Holm K, Niebuhr A, Eiberg H, Tommerup N, Hougaard S, Skovgaard Poulsen H, Spang-Thomsen M and Norgaard P (1997) Loss of the retinoblastoma-related p130 protein in small cell lung carcinoma. *Proc Natl Acad Sci USA* **94**: 6933–6938

Howard CM, Claudio PP, Gallia GL, Gordon J, Giordano GG, Hauck WW, Khalili K and Giordano A (1998) Retinoblastoma-related protein pRb2/p130 and suppression of tumor growth in vivo. *J Natl Cancer Inst* **90**: 1451–1460

Leoncini L, Bellan C, Cossu A, Claudio PP, Lazzi S, Cinti C, Ceverini G, Megha T, Laurini L, Luzi P, Orcioni GF, Piccioli M, Pileri S, Giardino C, Tosi P and Giordano A (1999) Retinoblastoma-related p107 and pRb2/p130 proteins in malignant lymphomas: distinct mechanisms of cell browth control. *Clin Cancer Res* **5**: 4065–4072

Li S-F, Shiozawa T, Nakayama K, Nikaido T and Fujii S (1996) Stepwise abnormality of sex steroid hormone receptors, tumor suppressor gene products (p53 and Rb), and cyclin E in uterine endometroid carcinoma. *Cancer* **77**: 321–329

Linardopoulos S, Papadakis E, Delakas D, Theodosiou V, Cranidis A and Spandidos DA (1993) Human lung and bladder carcinoma tumors as compared to their adjacent normal tissue have elevated AP-1 activity associated with the retinoblastoma gene promoter. *Anticancer Research* **13**: 257–262

Mayol X, Garriga J and Grana X (1996) Glcyclin/CDK-independent phosphorylation and accumulation of p130 during the transition from G1 to G0 lead to its association with E2F-4. *Oncogene* **13**: 237–246

Miller WR and Langdon SP (1997) Hormonal, growth factor, and cytokine control of breast cancer. In *Biology of female cancers*, Langdon SP, Miller WR and Berchuck A (eds) pp. 43–60. CRC Press: New York

Mori A, Higashi H, Hoshikawa Y, Imamura M, Asaka M and Hatakeyama M (1999) Granulocytic differentiation of myeloid progenitor cells by p130, the retinoblastoma tumor suppressor homologue. *Oncogene* **18**: 6209–6221

Mulligan G and Jacks T (1998) The retinoblastoma gene family: cousins with overlapping interests. *TIG* **14**: 223–229

Nishitani J, Nishinaka T, Cheng C-H, Rong W, Yokoyama KK and Chiu R (1999) Recruitment of the retinoblastoma protein to c-jun enhances transcription activity mediated through the AP-1 binding site. *J Biol Chem* **274**: 5454–5461

Pupa SM, Howard CM, Invernizzi AM, DeVecchi R, Giani C, Claudio PP, Colnaghi MI, Giordano A and Menard S (1999) Ectopic expression of pRb2/p130 suppresses the tumorigenicity of the c-erbB-2-overexpressing SKOV tumor cell line. *Oncogene* **18**: 651–656

Satyaswaroop PG and Tabibzadeh S (1997) Endometrial carcinoma: steroid hormones, growth factors, and cytokines. In *Biology of female cancers* Langdon, SP, Miller WR and Berchuck A (eds) pp. 193–203. CRC Press: New York

Schlott T, Reimer S, Jahns A, Ohlenbusch A, Ruschenburg I, Nagel H and Droese M (1997) Point mutations and nucleotide insertions in the mdm2 zinc finger structure of human tumours. *J Pathol* **182**: 54–61

Susini T, Baldi F, Howard CM, Baldi A, Taddei G, Massi D, Rapi S, Savino L, Massi G and Giordano A (1998) Expression of the retinoblastoma-related gene Rb2/p130 correlates with clinical outcome in endometrial cancer. *J Clin Oncol* **16**: 1085–1093

Tanaka N, Odajima T, Nakano T, Kimijima Y, Yamada S, Ogi K and Kohama G (1999) Immunohistochemical investigation of new suppressor oncogene p130 in oral squamous cell carcinoma. *Oran Oncol* **35**: 321–325

Tang Z, Treilleux I and Brown M (1997) A transcriptional enhancer required for the differential expression of the human estrogen receptor in breast cancers. *Mol Cell Biol* **17**: 1274–1280

Udvadia AJ, Rogers KT, Higgins PDR, Murata Y, Martin KHH, PA and Horowitz JM (1993) SP-1 binds promoter elements regulated by the RB protein and SP-1-mediated transcription is stimulated by RB coexpression. *Proc Natl Acad Sci USA* **90**: 3265–3269

Weinberg RA (1995) The retinoblastoma protein and cell cycle control. *Cell* **81**: 323–330

Yeung RS, Bell DW, Testa JR, Mayol X, Baldi A, Grana X, Klinga-Levan K, Knudson AG and Giordano A (1993) The retinoblastoma-related gene, Rb2 maps to human chromosome 16q12 and rat chromosome 19. *Oncogene* **8**: 3465–3468

*© 2001 Cancer Research Campaign*
